# Wheezing Characteristics and Predicting Reactivity to Inhaled β2-Agonist in Children for Home Medical Care

**DOI:** 10.3389/fped.2021.667094

**Published:** 2021-10-01

**Authors:** Chizu Habukawa, Naoto Ohgami, Takahiro Arai, Haruyuki Makata, Tomoki Nishikido, Morimitsu Tomikawa, Katsumi Murakami

**Affiliations:** ^1^Department of Pediatrics, Minami Wakayama Medical Center, Tanabe, Japan; ^2^Technology Development HQ, Omron Healthcare Co., Ltd., Muko, Japan; ^3^Arai Pediatric Clinic, Yamagata, Japan; ^4^Makata Pediatrics and Allergy Clinic, Yamaguchi, Japan; ^5^Osaka Women's and Children's Hospital, Izumi, Japan; ^6^Odasaga Pediatrics and Allergy, Sagamihara, Japan; ^7^Department of Psychosomatic Medicine, Sakai Sakibana Hospital, Sakai, Japan

**Keywords:** wheezing, young children, lung sound analysis, β2-agonists, wheeze analysis

## Abstract

**Background:** Given that wheezing is treated with inhaled β2-agonists, their effect should be reviewed before the condition becomes severe; however, few methods can currently predict reactivity to inhaled β2-agonists. We investigated whether preinhalation wheezing characteristics identified by lung sound analysis can predict reactivity to inhaled β2-agonists.

**Methods:** In 202 children aged 10–153 months, wheezing was identified by auscultation. Lung sounds were recorded for 30 s in the chest region on the chest wall during tidal breathing. We analyzed the wheezing before and after β2-agonist inhalation. Wheezing was displayed as horizontal bars of intensity defined as a wheeze power band, and the wheezing characteristics (number, frequency, and maximum intensity frequency) were evaluated by lung sound analysis. The participants were divided into two groups: non-disappears (wheezing did not disappear after inhalation) and disappears (wheezing disappeared after inhalation). Wheezing characteristics before β2-agonist inhalation were compared between the two groups.

The characteristics of wheezing were not affected by body size. The number of wheeze power bands of the non-responder group was significantly higher than those of the responder group (*P* < 0.001). The number of wheeze power bands was a predictor of reactivity to inhaled β2-agonists, with a cutoff of 11.1. The 95% confidence intervals of sensitivity, specificity, and positive and negative predictive values were 88.8, 42, 44, and 81.1% (*P* < 0.001), respectively.

**Conclusions:** The number of preinhalation wheeze power bands shown by lung sound analysis was a useful indicator before treatment. This indicator could be a beneficial index for managing wheezing in young children.

## Introduction

In the medical field, technical innovation has engendered telemedicine and home-based therapy; however, the practical use of these technologies has been limited. For respiratory diseases, lung sounds represent simple physical data, which have no value by themselves and are only clinically important when evaluated by a physician (1–3).

Appropriate judgment of lung sound data by specialists is required, especially when determining the patient's response to treatment, which constitutes important information in telemedicine. Acute exacerbation typically occurs at night and needs to be treated promptly; therefore, caregivers need to cope with the symptoms of this acute exacerbation. For parents, properly managing these exacerbations in young children is challenging, especially in infants. At present, there is no specific tool or criterion for treating these exacerbations.

Wheezing is a typical sign of respiratory exacerbation in individuals of all ages. Wheezing that occurs repeatedly over prolonged periods is an important factor in the diagnosis of bronchial asthma. Some young children with recurrent wheezing do not exhibit asthma (4, 5). Wheezing episodes in young children should initially be treated with inhaled short-acting beta-2 (β2) agonists, regardless of whether a clinical diagnosis of asthma has been made (6). According to international guidelines, initial treatment provided at home should comprise an inhaled β2-agonist, and the effect should be reviewed before the condition becomes severe (6). Physicians usually judge reactivity of inhaled β2-agonist by whether wheezing sounds disappear or do not disappear after inhaling the β2-agonist. Accurately evaluating a child's reactivity to inhaled β2-agonists at home is therefore challenging for parents/caregivers. There are currently few methods for predicting the effects of inhaled β2-agonists on wheezing in young children.

Wheezing is an important physical sign of worsening respiratory conditions that can be auscultated using a stethoscope (7–9). Recent developments in signal processing methods have improved the extraction of physiologically and clinically relevant information from a lung sound analysis (10–12), a noninvasive method that does not require the infant's cooperation and is useful in objectively evaluating wheezing. A number of studies have evaluated the severity of airway obstruction by assessing the particular characteristics of wheezing (13, 14).

However, wheezing characteristics have not yet been adequately analyzed to establish their association with reactivity to inhaled β2-agonists in exacerbations. Our aim is to develop a home medical device to evaluate the wheezing condition. In this study, we investigated (as an objective index) whether preinhalation wheezing characteristics, detected by lung sound analysis, could predict reactivity to inhaled β2-agonists in children, including infants.

## Materials and Methods

### Participants

All participants were outpatients from the Arai Pediatric Clinic, Makata Pediatrics and Allergy Clinic, Osaka Women's and Children's Hospital, Odasada Pediatrics and Allergy, or Minami Wakayama Medical Center in Japan, who were brought to each of these hospitals to treat recurrent wheezing, cough, and dyspnea. All participants had more than 3 episodes of wheezing and were diagnosed with asthma according to the Japanese pediatric guidelines for the treatment and management of asthma (6). The exclusion criteria included the presence of respiratory syncytial virus infection, human metapneumovirus infection, chronic wheezing diseases, laryngomalacia, whooping cough, immunodeficiency, and cardiac and neonatal pulmonary problems. These diagnoses were evaluated by a pediatrician who specializes in childhood respiratory and allergic diseases.

The required number of samples was 41, with an AUC of 0.8, a power of 0.95, and a one-sided test (*R* version 3.4.1 software). The study population comprised 202 pediatric outpatients (median age, 19.9 months; range, 10–153 months; male/female ratio, 107/95). Written informed consent was obtained from the parents or legal guardians of all participants, and the study protocol was approved by the Minami Wakayama Medical Center's ethics committee [approval number 2016–22 (2)].

### Study Design

All participants exhibited audible wheezing during tidal breathing, as observed by auscultation. Lung sounds were recorded for 30 s in the upper right anterior chest region at the second intercostal space and at the midclavicular line on the chest wall during tidal breathing. The lung sounds, including wheezing, were identified and independently confirmed by each specialist physician who recorded the wheezing according to previous methods (15). A total of 197 participants, for whom data were available, were examined to evaluate heart rate and oxygen saturation. Information regarding symptom exacerbation, including wheezing, coughing, heavy breathing, reduced activity, shortness of breath, and sleep disturbance was obtained from the parents or legal guardians.

Thereafter, all participants were treated with inhaled β2-agonists (10–30 μg of procaterol and 2.0 ml of saline) (6, 16). The study physicians auscultated and recorded the lung sounds simultaneously 15 min after the β2-agonist inhalation (12, 15). All participants were treated according to the Japanese pediatric guidelines for the treatment and management of asthma (6).

Wheezing was evaluated to identify changes before and after inhalation via auscultation and lung sound analysis. According to the reactivity to the inhaled β2-agonists, the participants were divided into two groups: non-disappear and disappear. Participants whose wheezing disappeared after inhaling β2-agonists were assigned to the disappear group, whereas those whose wheezing did not disappear after inhaling β2-agonists were assigned to the non-disappear group.

We compared the physical signs, including heart rate, oxygen saturation, and participant characteristics between the two groups. The wheezing characteristics before inhaling β2-agonists were compared between the two groups as described below.

### Sound Recording and Sound Analysis

**Figure 1:**
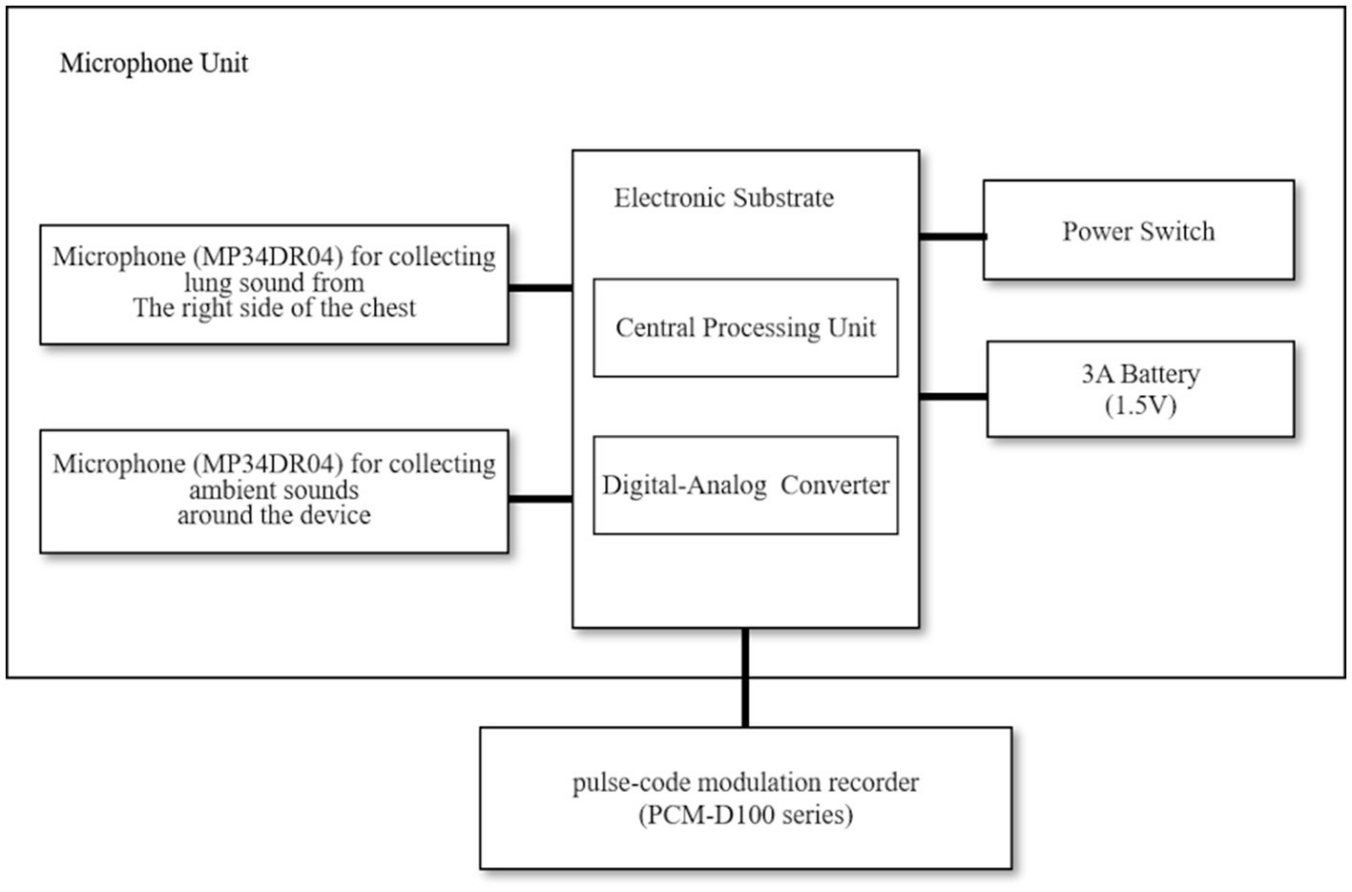
Block diagram showing the equipment used for lung sound recording.

Figure 1 shows a block diagram of the equipment employed in the lung sound recordings (17). The sound recording system consisted of a handheld assembled microphone unit (prototype device, Omron Healthcare Corporation, Ltd., Kyoto, Japan) and a pulse-code modulation recorder (PCM-D100 series, Sony Corporation, Tokyo, Japan). Two microphones (MP34DR04, STMicroelectronics, Geneva, Switzerland) were set in the microphone unit; one collected ambient sounds around the device, while the other collected lung sounds from the right side of the chest. We resampled the lung sounds at 44.1-kHz with 16-bit quantization, performing a 4,096-point fast Fourier transformation using sound analysis software (Adobe Audition CC 2018, Adobe Inc., San Jose, CA, United States).

**Figure 2:**
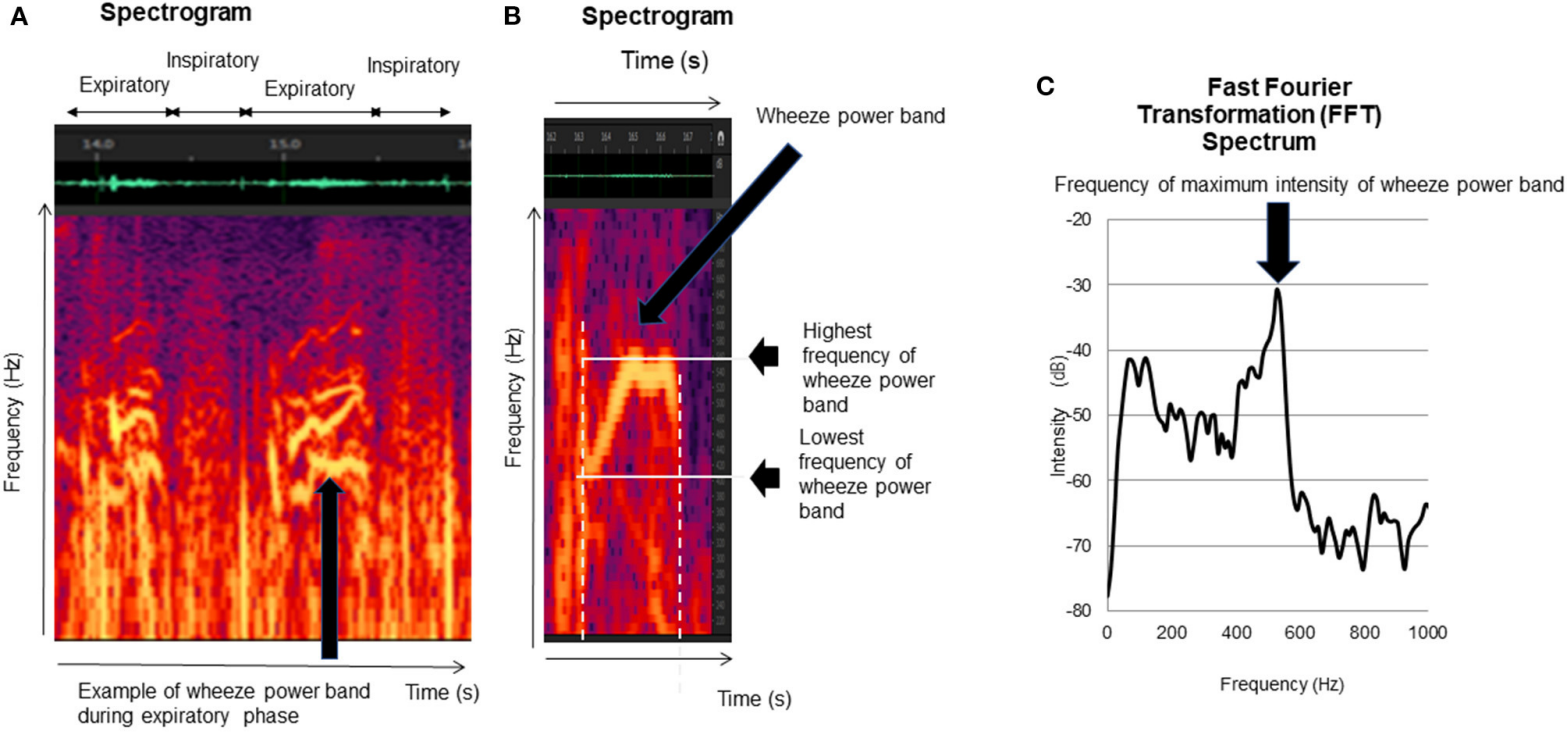
Wheezing indexes. The lung sound analysis displayed wheezes as horizontal bars of intensity with corresponding sharp peaks of power. The sharp peak of power was defined as a wheeze. The following indexes were calculated: (1) duration of wheeze power bands **(B)**, (2) number of wheeze bands per 30s **(B)**, (3) lowest frequency of wheeze power bands **(B)**, (4) highest frequency of wheeze power bands **(B)**, and (5) frequency of maximum intensity of wheeze power bands **(C)**.

Figure 2 shows a sample of the spectrogram and spectrum of the wheezing sounds (17). Prior to the sound analysis, engineering researchers and two trained physicians evaluated all recordings. The recorded lung sounds were reviewed to discriminate wheezes from other sounds, such as noises generated due to friction between the microphone and the participants' skin. We detected wheezing on the sound spectrograms as defined by the Computerized Respiratory Sound Analysis (CORSA) guidelines. A wheeze is defined as a continuous adventitious musical sound. Acoustically, it is characterized by periodic waveforms with a dominant frequency typically over 100 Hz and lasting over 100 ms (18).

### Wheezing Indexes

The lung sound analysis is displayed as horizontal bars of intensity with corresponding sharp peaks of power. The sharp peak of power was defined as a wheeze power band (Figure 2A) (17, 19–21). Wheezing was analyzed during both inspiratory and expiratory periods in each recorded file according to the six indexes explained below.

#### Number of Wheeze Power Bands per 30s

We counted the number of wheeze power bands per 30s in each file (17).

#### Lowest Frequency of Wheeze Power Bands

We calculated the lowest frequencies of all wheeze power bands as described above (Figure 2B) (17).

#### Highest Frequency of Wheeze Power Bands

We recorded the highest frequency that could be recognized on the spectrogram (Figure 2B) (17), as well as the highest frequencies of all wheeze power bands in each file, and calculated the mean frequencies of all wheeze power bands from the individual data.

#### Maximum Intensity Frequency of Wheeze Power Bands

We recorded the maximum intensity frequency of all wheeze power bands (20, 21) and calculated the mean frequency of the wheeze power bands' maximum intensity for each recorded file (Figure 2C) (17).

### Statistical Analysis

The correlation coefficients between age, weight, and height with sound variables were determined using Pearson's correlation coefficient.

We performed the statistical analyses using STATFLEX ver. 6.0 (Artec Co., Ltd., Osaka, Japan). The wheezing indexes and participant characteristics are presented as the mean ± standard deviation. We compared the participant characteristics and wheezing indexes between the two groups using an unpaired *t*-test.

We calculated the sensitivity (true-positive rate), specificity (true-negative rate), positive predictive value, negative predictive value, and positive and negative likelihood ratios (the probability of symptom exacerbation according to the wheezing index cutoff value), and we used a receiver operating characteristic curve to describe the relationship between the sensitivity and specificity of the various cutoff values (wheezing index) as reactivity to the inhaled β2-agonists.

We also calculated the area under the curve (AUC) for all possible cutoff values of the highest frequency of the wheeze power bands. *P* < 0.05 were considered statistically significant. The confidence interval of the data analyzed was 95%.

## Results

### Differences in Participant Characteristics

Table 1 lists the participant characteristics and respiratory statuses of the two groups. Except for heart rate, there were no significant differences between the two groups.

**Table 1:** Participants' characteristics and respiratory statuses of the two groups.

	**Disappear Group (*n* = 91)**	**Nondisappear Group (*n* = 111)**	***P-value***
Age, month (Range)	51.5 ± 41.2 (10~151)	48.8 ± 34.0 (10~153)	0.62
Sex (M/F)	43./48	64./47	0.09
Height, cm	98.6 ± 21.8	97.4 ± 18.9	0.70
Weight, kg	16.4 ± 8.2	16.4 ± 8.4	0.87
Food allergy (positive %)	81.0%	93.8%	0.27
House dust and/or Mice allergy (positive %)	87.5%	63.3%	0.18
Total IgE, IU/ml	944.7 ± 815.5	971.8 ± 109.1	0.37
SaO_2_, %	98.0 ± 1.4	97.7 ± 1.5	0.30
Heart rate, bpm	107.3 ± 26.1	121.8 ± 17.8	^*^ 0.008
Respiratory rate, /min	31.0 ± 14.1	30.6 ± 10.8	0.86

* *P-values of < 0.01 was considered statistically significant. Values are presented as mean ± standard deviation. IgE, immunoglobulin E; SaO_2_, oxygen saturation*.

### Correlations of the Wheezing Indexes With Age, Height, and Weight

All the wheezing indexes were not significantly affected by age, height, and weight (Table 2). The respiratory cycle was not significantly correlated with all the wheezing indexes.

**Table 2:** *P*-values of correlations of the wheezing indexes with age, height, and weight.

	**Age (month)**	**Height(cm)**	**Weight (kg)**	**Respiratory Rate(breaths /min)**
Number of wheeze power bands per 30 s	0.04	0.04	0.08	0.17
Highest frequency of wheeze power bands (Hz)	0.07	0.03	0.01	0.007
Lowest frequency of wheeze power bands (Hz)	0.18	0.14	0.14	0.04
Maximum intensity frequency of the wheeze power bands (Hz)	0.18	0.13	0.11	0.04

#### Comparison of the Wheeze Power Band Duration

No significant differences were observed in the duration of wheeze power bands between the two groups (*P* = 0.09). The duration of the wheeze power bands in the disappear group was 338.9 ± 202.7 ms, whereas that of the non-disappear group was 398.3 ± 205.9 ms.

#### Comparison of the Highest Frequency of the Wheeze Power Bands

**Figure 3:**
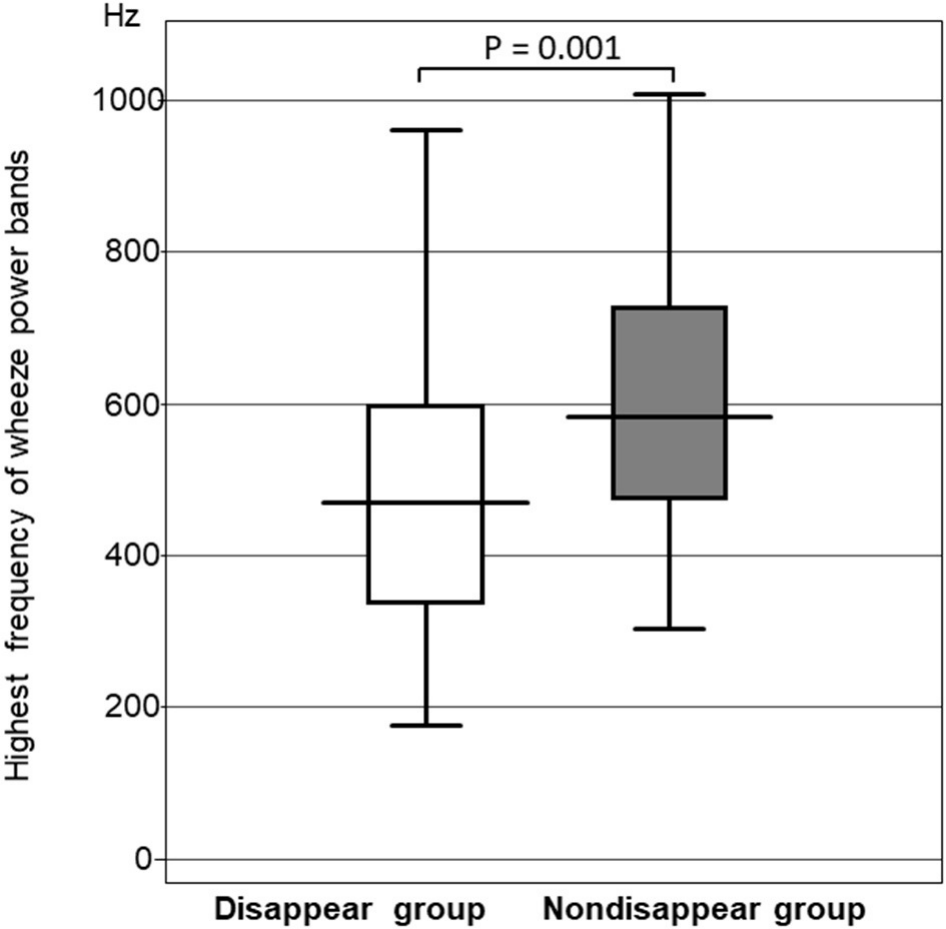
Comparison of the highest frequency of wheeze power bands between the two groups.

The highest frequency of the wheeze power bands was significantly higher in the non-disappear group than in the disappear group (*P* = 0.0014). The highest frequency of the wheeze power bands in the non-disappear group was 592.6 ± 223.4 Hz, whereas that of the disappear group was 460.8 ± 220.1 Hz (Figure 3).

#### Comparison of the Lowest Frequency of the Wheeze Power Bands

No significant differences were observed between the two groups in the lowest frequency of the wheeze power bands (*P* = 0.23). The lowest frequency of the wheeze power bands in the non-disappear group was 235.2 ± 106.8 Hz, whereas that of the disappear group was 210.3 ± 144.6 Hz.

#### Comparison of the Maximum Intensity Frequency of the Wheeze Power Bands

**Figure 4:**
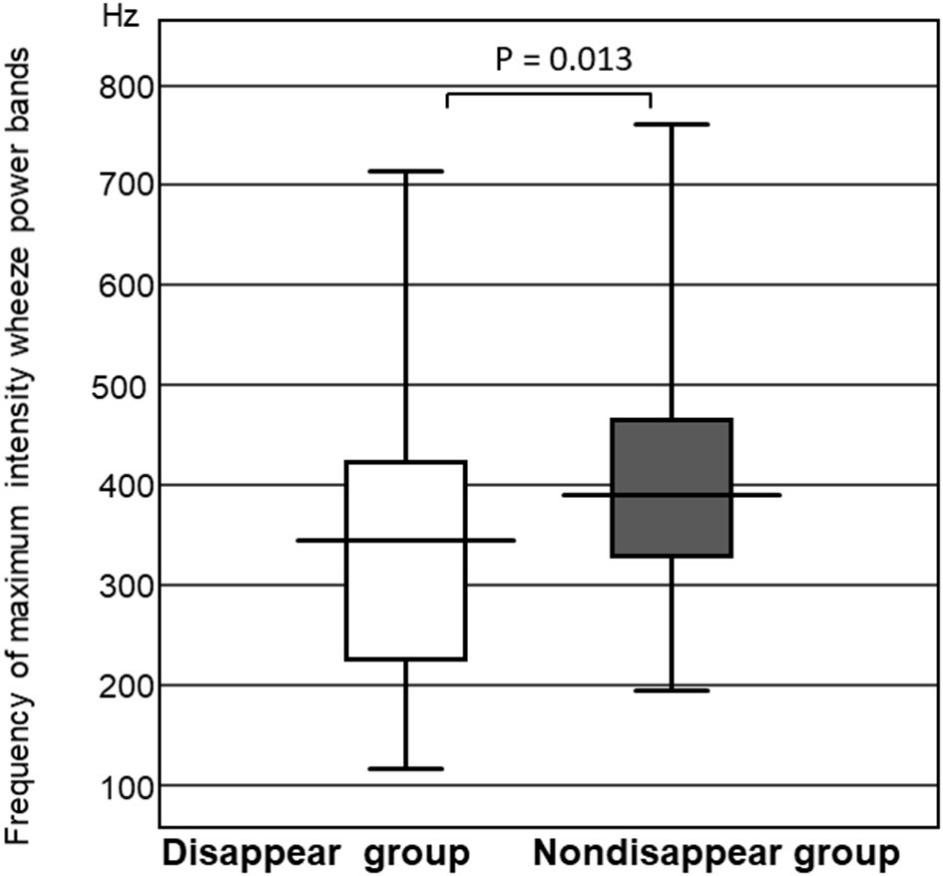
Comparison of the frequency of maximum intensity wheeze power bands between the two groups.

The frequency of the wheeze power bands was significantly higher in the non-disappear group than that of the disappear group (*P* = 0.013). The maximum intensity frequency of the wheeze power bands in the non-disappear group was 396.1 ± 136.7 Hz, whereas that of the disappear group was 331.3 ± 175.7 Hz (Figure 4).

#### Comparison of the Number of Wheeze Power Bands per 30s

**Figure 5:**
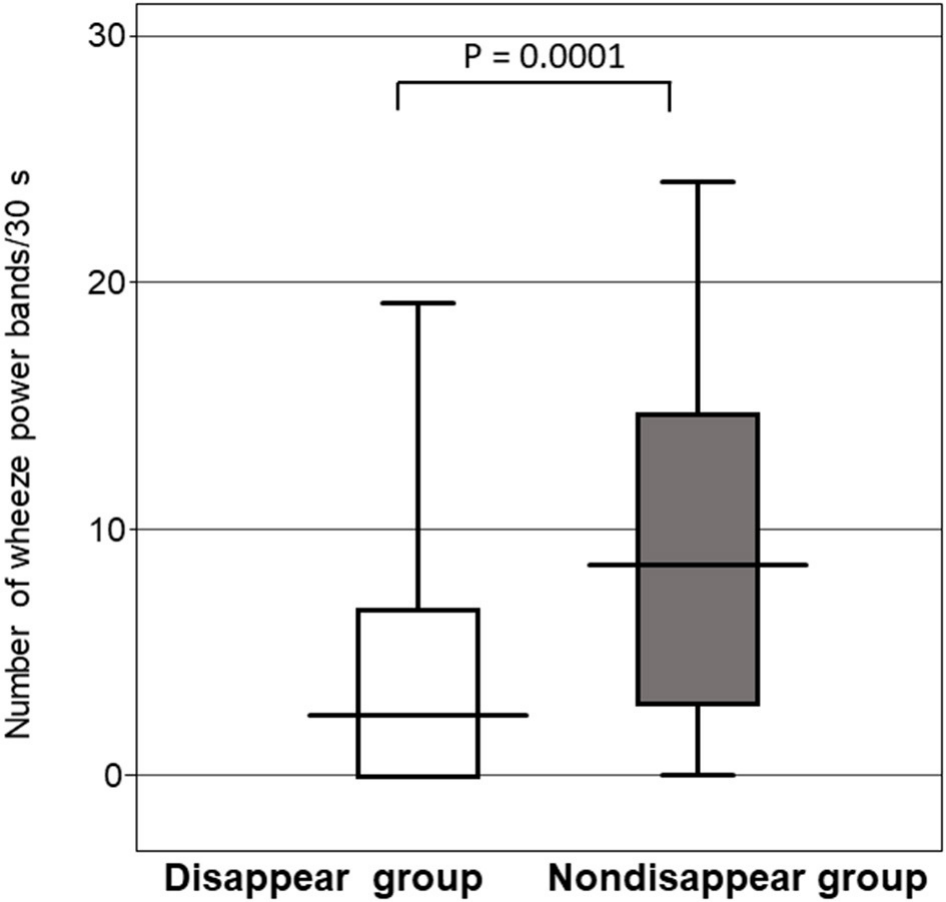
Comparison of the number of wheeze power bands per 30 s between the two groups. Horizontal line indicates the response to the inhaled β2-agonist, and the error bars indicate the standard deviation.

Figure 5 shows that the number of wheeze power bands per 30s for the non-disappear group was larger than that of the disappear group (*P* = 0.0001). The number of wheeze power bands per 30s was 9.8 ± 7.3 for the non-disappear group and 4.1 ± 5.3 for the disappear group.

### Receiver Operating Characteristic Curve Analysis in Response to Inhaled β2-Agonists With Respect to the Number of Wheeze Power Bands per 30s

**Figure 6:**
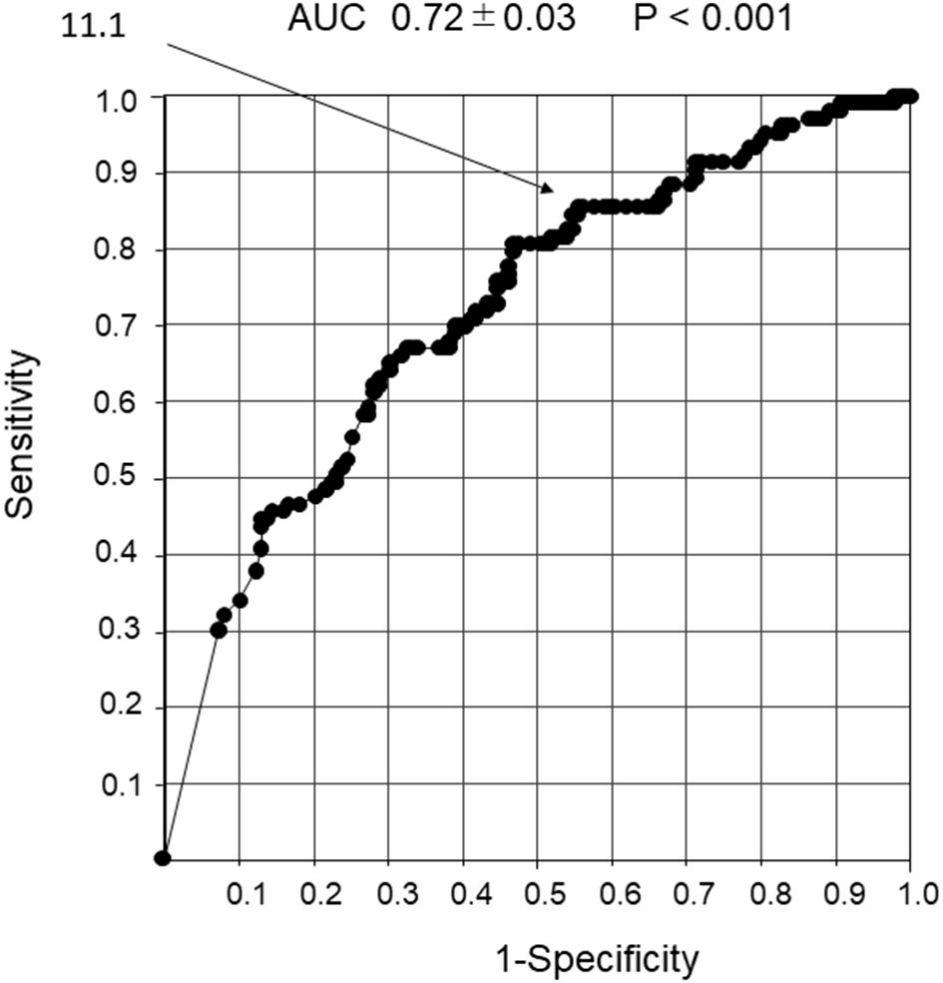
Receiver operator characteristic curve analysis in response to the β2-agonist inhalation with the number of wheeze power bands per 30 s. Detection of non-response to inhaled β2-agonist using the number of wheeze power bands per 30 s. An ROC curve analysis was performed to determine the response to inhaled β2-agonist with the number of wheeze power bands per 30 s. The cutoff value for the number of wheeze power bands per 30 s that could predict reactivity to the inhaled β2-agonist was 11.1.

The cutoff value for the number of wheeze power bands per 30 s that could predict a response to the inhaled β2-agonists was 11.1. The 95% confidence intervals of sensitivity, specificity, positive and negative predictive values, and positive and negative likelihood ratios were 88.8, 42, 44, and 81.1%, and 1.53 and 0.27 (*P* < 0.001), respectively, with an AUC of 0.72 ± 0.03 (Figure 6).

## Discussion

In the present study, the characteristics of preinhalation wheezing could predict reactivity to inhaled β2-agonists. We found that the number of preinhalation wheeze power bands was a predictor of reactivity to inhaled β2-agonists and a useful indicator of prolonged exacerbation risk. None of the wheezing characteristics were affected by height, weight, or age. Noninvasively and objectively predicting the reactivity to inhaled β2-agonists before administering treatment is crucial for managing wheezing in children. Understanding the differences in the characteristics of wheezing can be beneficial for administering treatment and managing children with asthma for remote medical care that children cannot go to hospital when they afraid catch some virus infection using home medical device which we developed (22).

Wheezing is a continuous adventitious lung sound that is superimposed on breathing sounds. According to the definitions in the present CORSA guidelines, the maximum frequency of a wheeze is typically >100 Hz, and its duration is >100 ms (19–21). Baughman and Loudon reported that wheezing was also associated with a peak in the signal and that peak amplitude constituted a criterion for classifying the sound as a wheeze. Wheezing was displayed as horizontal bars of intensity with corresponding sharp peaks of power, known as wheeze power bands (23). Using lung sound analysis, we found that the wheezing of non-disappear group had certain wheeze power band characteristics, such as higher number, higher frequency, and higher frequency at maximum intensity.

Several studies have reported the association between wheezing characteristics and lung function, with one study showing that expiratory wheezing had high respiratory resistance in infants (14).

For the frequency and intensity of wheezing, Shim and Williams found that high frequency, louder, and longer wheezing were associated with a lower peak expiratory flow rate. Loudness and high frequency wheezing are associated with more severe obstruction (24). The degree of bronchospasm (abnormal muscle contraction in the bronchi walls, causing airway obstruction) is related to the frequency of wheezing sound signals, rather than the wheezing intensity (25). Our data correspond with this suggestion.

Based on the number of wheeze power bands, wheezing is divided into two categories according to international guidelines' best-known signs of airway obstruction: monophonic and polyphonic (9). Wheezing is considered monophonic when only one pitch is heard and polyphonic when multiple frequencies are simultaneously perceived. Polyphonic wheezing involves more severe bronchial constriction than monophonic wheezing (3, 17, 18). Wheezing is considered monophonic when there is only a peak intensity of one frequency and polyphonic when numerous peak intensities of varying frequencies are perceived simultaneously by the lung sound analysis. In other words, monophonic wheezes have one wheeze power band, while polyphonic have multiple wheeze power bands, thereby indicating that polyphonic wheezing involves more severe bronchial constriction that monophonic wheezing (9, 24, 25). The number of wheeze bands in patients with polyphonic wheezing was greater than that of patients with monophonic wheezing. Moreover, patients with more wheeze bands have a greater risk of exacerbation (9, 19–21).

In our study, the non-disappear group had more wheeze power bands than the disappear group. In other words, the patients in the non-disappear group believed that they had a more severe airway obstruction. The study subjects were young children with a mean age of 19.9 months who had bronchial asthma and viral lower respiratory tract infections associated with asthma. In our subjects, the cause of airway obstruction could be a combination of airway inflammation and airway spasm, and the treatment response to bronchodilator inhalation therapy might have been different.

For home treatment, parents use bronchodilator inhalation to prevent exacerbations even when it is difficult to identify whether the wheezing is due to an asthma attack or a viral infection associated with asthma. The cutoff value for wheeze power bands was set to have low specificity but high sensitivity so that parents do not overlook signs of deterioration. Thus, wheeze power bands may be a useful index for avoiding the risk of future deterioration of respiratory status because the therapeutic effect can be predicted before inhalation of bronchodilators regardless of the cause of wheezing.

Gavriely et al. employed a wheeze-detection device for overnight nocturnal monitoring to assess asthma activity in symptomatic school-aged children (26). Although wheezing monitoring is useful for asthma management, it is difficult to record wheezing overnight in young children, particularly in infants, because they cannot endure being attached to a monitor for a long period, and it is difficult for the family to manage and prevent the microphone from becoming dislodged. It is therefore important to perform the examination in young children within a short period. Furthermore, it would be more useful for the family to have knowledge of the characteristics of preinhalation wheezing so they can predict reactivity to inhaled β2-agonists. However, it should be noted that this study had the limitation that the participants with severe airway obstruction did not demonstrate any audible lung sounds (known as “silent chest”).

Wheezing characteristics are presumably associated with bronchial obstruction. To date, there have been no reports regarding how the preinhalation characteristics of wheezing can predict reactivity to inhaled β2-agonists. The number of preinhalation wheeze power bands assessed by lung sound analysis was a useful indicator for predicting reactivity to inhaled β2-agonists of airway obstruction in younger children. The study subjects all had mild asthma attacks. As shown in Table 1, SaO_2_ did not decrease in the non-disappear group, and only heart rate increased as compared with that in the disappear group. However, no significant differences were found in SaO_2_ and respiratory rates during the stable respiratory state. The number of power band was a highly sensitive index predictive exacerbation even in mild attacks with increased heart rate and before decreased SaO_2_. This information can be obtained before treatment using a noninvasive method and a 30-s assessment. The results could be useful for managing wheezing in young children with mild to moderate attack, by physicians, parents, and legal guardians.

Lung sound analysis can noninvasively detect detailed characteristics of wheezing in young children, including infants. The number of wheeze power bands before inhalation may be a predictor of responsiveness to bronchodilator inhalation, regardless of asthma or virus infection in children, including infants.

In home treatment, even when the cause of wheezing is unclear, either asthma attack or concomitant viral infection, the therapeutic effect of bronchodilator inhalation can be predicted and considered as an index for avoiding the risk of exacerbation.

## Data Availability Statement

The raw data supporting the conclusions of this article will be made available by the authors, without undue reservation.

## Ethics Statement

The studies involving human participants were reviewed and approved by Yoshinori Nakamura, Minami Wakayama Medical Center's ethics committee. Written informed consent to participate in this study was provided by the participants' legal guardian/next of kin. Written informed consent was obtained from the individual(s), and minor(s)' legal guardian/next of kin, for the publication of any potentially identifiable images or data included in this article.

## Author Contributions

CH was the guarantor of the manuscript. CH and KM conceived, designed the study, analyzed, and interpreted the data. CH, TA, HM, TN, and MT collected the data. CH prepared the manuscript. All authors approved the final version of the manuscript. Omron Healthcare Co., Ltd. had no such involvement.

## Funding

The authors declare that this study received funding from Omron Healthcare Co., Ltd. The funder was not involved in the study design, collection, analysis, interpretation of data, the writing of this article or the decision to submit it for publication.

## Conflict of Interest

CH and KM received research grant from the Omron Health Care Corporation. NO is an employee of Omron Healthcare Co., Ltd. The remaining authors declare that the research was conducted in the absence of any commercial or financial relationships that could be construed as a potential conflict of interest.

## Publisher's Note

All claims expressed in this article are solely those of the authors and do not necessarily represent those of their affiliated organizations, or those of the publisher, the editors and the reviewers. Any product that may be evaluated in this article, or claim that may be made by its manufacturer, is not guaranteed or endorsed by the publisher.

## References

[1] Aliverti A. Wearable technology: role in respiratory health and disease. Breathe. (2017) 13:e27–36. 10.1183/20734735.008417 28966692 PMC5621614

[2] Zhang J Ser W Yu J Zhang TT. A novel wheeze detection method for wearable monitoring systems. In: International Symposium on Intelligent Ubiquitous Computing and Education (2009). Chengdu: IEEE Computer Society (2009). 331–4. 10.1109/IUCE.2009.66

[3] Taplidou SA Hadjileontiadis LJ Kitsas IK Panoulas KI Penzel T Gross V. On applying continuous wavelet transform in wheeze analysis. In: The 26th Annual International Conference of the IEEE Engineering in Medicine and Biology Society. San Francisco, CA: IEEE (2004) 2:3832–5. 17271131 10.1109/IEMBS.2004.1404073

[4] McFadden ER Kiser R DeGroot WJ. Acute bronchial relations between clinical and physiologic manifestations. N Engl J Med. (1973) 288:221–5. 10.1056/NEJM197302012880501 4682217

[5] Godfrey S Edwards RHT Campbell EJM Armitage P Oppenheimer EA. Repeatability of physical signs in airway obstruction. Thorax. (1969) 24:4–9. 10.1136/thx.24.1.4 4884174 PMC471914

[6] Arakawa H Hamasaki Y Kohno Y Ebisawa M Kondo N Nishima S. Japanese guidelines for childhood asthma 2017. Allergol Int. (2017) 66:190–204. 10.1016/j.alit.2016.11.003 28108245

[7] American Thoracic Society Committee on Pulmonary Nomenclature. Am Thorac Soc News. San Francisco, CA: American Thoracic Society Committee on Pulmonary Nomenclature (1977) 3:6.

[8] Murphy RL. In defense of the stethoscope. Respir Care. (2008) 53:355−69. 18291053

[9] Forgacs P. The functional basis of pulmonary sounds. Chest. (1978) 73:399–405. 10.1378/chest.73.3.399 630938

[10] Habukawa C Murakami K Endoh M Horii N Nagasaka Y. Treatment evaluation using lung sound analysis in asthmatic children. Respirology. (2017) 22:1564–9. 10.1111/resp.13109 28722791

[11] Shimoda T Obase Y Nagasaka Y Nakano H Kishikawa R Iwanaga T. Lung sound analysis is useful for monitoring therapy in patients with bronchial asthma. J Allerg Clin Immunol. (2017) 27:246–51. 10.18176/jiaci.0132 28731412

[12] Habukawa C Murakami K Endoh M Yamada M Horii N Nagasaka Y. Evaluation of airflow limitation using a new modality of lung sound analysis in asthmatic children. Allergol Int. (2015) 64:84–9. 10.1016/j.alit.2014.08.006 25572561

[13] Pasterkamp H Brand PL Everard M Garcia-Marcos L Melbye H Priftis KN. Towards the standardisation of lung sound nomenclature. Eur Respir J. (2016) 47:724–32. 10.1183/13993003.01132-2015 26647442

[14] Fischer HS Puder LC Wilitzki S Usemann J Bührer C Godfrey S. Relationship between computerized wheeze detection and lung function parameters in young infants. Pediatr Pulmonol. (2016) 51:402–10. 10.1002/ppul.23310 26360639

[15] Bentur L Beck R Berkowitz D Hasanin J Berger I Elias N. Adenosine bronchial provocation with computerized wheeze detection in young infants with prolonged cough: correlation with long-term follow-up. Chest. (2004) 126:1060–5. 10.1378/chest.126.4.1060 15486364

[16] Marques A Oliveira A Jacome C. Computerized adventitious respiratory sounds as outcome measures for respiratory therapy: a systemic review. Respir Care. (2014) 5:765–76. 10.4187/respcare.02765 24046460

[17] Habukawa C Ohgami N Matsumoto N Hashino K Asai K Sato T. Wheeze sound characteristics are associated with nighttime sleep disturbances in younger children. Asia Pac Allergy. (2020) 10:e26. 10.5415/apallergy.2020.10.e26 32789111 PMC7402944

[18] Sovijarvi ARA Dalmasso F Vanderschoot J Malmberg LP Righini G Stoneman AAT. Definition of terms for applications of respiratory sounds. Eur Respir Rev. (2000) 10:597–610.

[19] Sovijärvi ARA Malmberg LP Charbonneau G Vanderschoot J Dalmasso F Sacco C. Characteristics of breath sounds and adventitious respiratory sounds. Eur Respir Rev. (2000) 10:591–6.

[20] Meslier N Charbonneau G Racineux JL. Wheezes. Eur Respir J. (1995) 8:1942–8. 10.1183/09031936.95.08111942 8620967

[21] Sovijarvi AR Vanderschoot J Earis JE. Standardization of computerized respiratory sound analysis. Eur Respir Rev. (2000) 10:585.

[22] Chizu Habukawa Naoto Ohgami Naoki Matsumoto Kenji Hashino Kei Asai Tetsuya Sato. A wheeze recognition algorithm for practical implementation in children. PLoS ONE. (2020) 15:e0240048. 10.1371/journal.pone.0240048 33031408 PMC7544038

[23] Baughman RP Loudon RG. Lung-sound analysis for continuous evaluation of airflow obstruction in asthma. Chest. (1985) 88:364–8. 10.1378/chest.88.3.364 4028846

[24] Shim CS Williams MH. Relationship of wheezing to the severity of obstruction in asthma. Arch Intern Med. (1983) 143:890−2. 6679232

[25] Baughman RP Loudon RG. Quantitation of wheezing in acute asthma. Chest. (1984) 86:718–22. 10.1378/chest.86.5.718 6488909

[26] Bentur L Beck R Shinawi M Naveh T Gavriely N. Wheeze monitoring in children for assessment of nocturnal asthma and response to therapy. Eur Respir J. (2003) 21:621–6. 10.1183/09031936.03.00036302 12762346

